# *DICER1*-Mutated Botryoid Fibroepithelial Polyp of the Parotid Duct: Report of the First Case

**DOI:** 10.1007/s12105-021-01364-y

**Published:** 2021-07-19

**Authors:** Ramona Erber, Raimund Preidl, Robert Stoehr, Florian Haller, Arndt Hartmann, Marco Kesting, Abbas Agaimy

**Affiliations:** 1 Institute of Pathology, Friedrich-Alexander University Erlangen-Nürnberg (FAU), University Hospital Erlangen (UKER), Krankenhausstrasse 8-10, 91054 Erlangen, Germany; 2 Comprehensive Cancer Center Erlangen-EMN (CCC ER-EMN), Erlangen, Germany; 3 Department of Oral and Maxillofacial Surgery, Friedrich‐Alexander University Erlangen‐Nürnberg (FAU), University Hospital Erlangen (UKER), Erlangen, Germany

**Keywords:** *DICER1*, Mutations, Salivary gland, Parotid duct, Fibroepithelial polyp, Botryoid polyp

## Abstract

*DICER1*, a member of the ribonuclease III family, is involved in the biogenesis of microRNAs and, hence, it influences gene expression regulation. *DICER1* germline (associated with the inherited *DICER1* syndrome) or somatic mutations have been linked to tumorigenesis in histogenetically diverse benign and malignant neoplasms in different organs including pleuropulmonary blastoma, cystic nephroma, embryonal rhabdomyosarcoma, nasal chondromesenchymal hamartoma, poorly differentiated thyroid carcinoma, thyroblastoma, intracranial sarcoma and gonadal Sertoli-Leydig cell tumors in addition to others. Moreover, rare botryoid (giant) fibroepithelial polyps may harbor this mutation. Herein, we describe the first reported case of a *DICER1-*mutated botryoid fibroepithelial polyp occurring within the parotid duct of a 65-year-old female who has no other features or family history of the DICER1 syndrome. Based on its distinctive morphology, we tested this lesion specifically for *DICER1* mutations and confirmed the presence of a pathogenic *DICER1* variant with a low allele frequency, consistent with a somatic mutation.

## Introduction

*DICER1,* located on the long arm of chromosome 14 (14q32.13, OMIM# 606241), encodes for Dicer protein which belongs to the ribonuclease III family and influences the activity of other genes by regulation of microRNA (miRNA) biogenesis [1]. *DICER1* mutations are involved in deregulation of miRNA and, hence, influence tumorigenesis [2]. During the last decade, *DICER1* mutations have been increasingly recognized to be associated with diverse benign and malignant neoplastic entities with distinctive morphological features that represent clues to an underlying *DICER1* mutation. While the majority of *DICER1* mutations represent germline variants in the setting of the inherited DICER1 syndrome, sporadic diseases with *DICER1* mutations have been recently recognized, albeit rare. Individuals affected by germline *DICER1* mutations tend to develop multinodular goiter [2], and have an increased risk of developing a variety of tumors, including pleuropulmonary blastoma [2], Wilms tumor (with low frequency) [3], cystic nephroma, embryonal rhabdomyosarcoma, nasal chondromesenchymal hamartoma [4], gynandroblastoma [5], esophageal polyps [6], medulloblastoma [7], and Sertoli-Leydig cell tumors of the ovary [2, 8]. To date, > 1136 *DICER1* variants (both germline and somatic) comprising protein truncating, in-frame deletions, and missense mutations have been described in > 808 individuals [2, 6, 8, 9]. Recently, we have described a botryoid fibroepithelial polyp of the urinary bladder carrying a *DICER1* mutation [10]. We herein describe the first reported case of a *DICER1*-mutated botryoid fibroepithelial polyp of the parotid duct.

### Clinical History

**Fig. 1:**
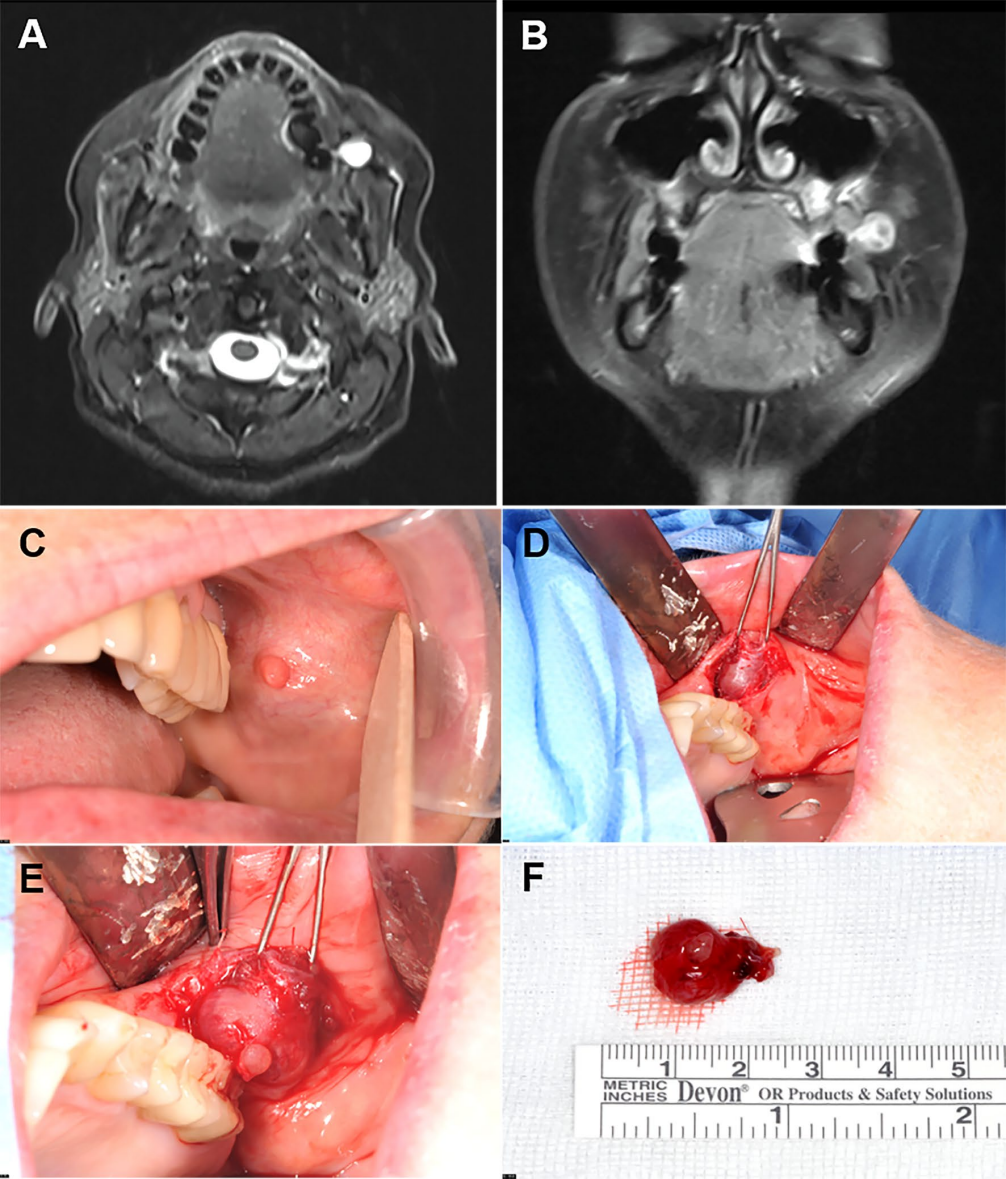
Preoperative MRI showed a mass in the left buccal region with hyperintense signaling in T2 sequence (**A**) and partially contrast-enhancing and hypointense signaling in T1 sequence (**B**). Clinically, the mass was closely associated with the papilla of the parotid duct (**C**). Intraoperatively, the duct was found to run through the lesion which required a cannulation of the residual duct after total resection with a reinsertion of the duct posterior to its original location (**D**, **E**). **F** Gross specimen of the resected mass showing a well circumscribed glistening surface

A 65- year- old woman presented with a progressively growing painless mass in her left buccal mucosa for 8 weeks. The mass was mobile and palpable in the region of the parotid duct next to the parotid papilla. There was no purulence or evidence of total parotid duct obstruction. The patient’s medical history was negative for neoplasms or other significant co-morbidities. Her family history revealed that the patient’s mother died of pancreatic cancer at age 56. Her father and three sisters had no cancer history. Preoperative MRI revealed a solid mass meassuring 1.3 × 1.0 × 0.9 cm located adjacent to the left masseter muscle with partial compression of the parotid duct. The mass was only partially contrast-enhancing and was hypointense in T1 sequence and hyperintense in T2 sequence (Fig. 1A, B).

Intraoperatively, the mass was well-demarkated from the surrounding soft tissue without infiltration. It was resected together with the adjacent parotid duct and the papilla with reinsertion of the parotid duct posterior to its original location (Fig. 1C-E). A splinting was fixed within the duct to maintain patency. The postoperative course was unremarkable, and the patient was released on the 3rd postoperative day.

## Material and Methods

The surgical specimen was fixed in formalin and embedded routinely for histopathological evaluation. Hematoxylin and eosin (H&E) stained slides were prepared for histopathological examination and immunohistochemical staining was performed on 1 µm thick sections using an automated platform (Benchmark Ultra, Ventana Medical Systems Inc., Tucson, Arizona, U.S.A.) and the following antibodies: CD34 (clone QBEND-10; 1:50, Immunotech), smooth muscle actin (clone 1A4, 1:400, Dako), desmin (clone D33, 1:50, Dako), MyoD1 (clone 5.8A, 1:50, Dako), myogenin (clone F5D, 1:50, Dako), S100 (clone 4C4.9, 1:3000, Zytomed), MDM2 (clone IF2, 1:50, Calbiochem), CDK4 (clone DCS-156, 1:100, Zytomed), Retinoblastoma-1 (clone G3-245, 1:100, BD Pharmingen), CD10 (clone 56C6, 1:20, Zytomed), STAT6 (clone S-20, 1:1000, Santa Cruz) and SATB2 (clone EPNCIR130A, 1:200, abcam).

### Molecular Testing

After careful manual microdissection, DNA was extracted from FFPE tumor tissue using the Maxwell^©^ 16 system (Promega, Madison, Wisconsin, USA) according to manufacturer’s instructions. *DICER1* sequence analysis was performed using the QIAseq Targeted Human Comprehensive Cancer Panel encompasing 160 cancer-related genes according to manufacturer’s instructions. Bioinformatic evaluation of the sequencing data, including variant calling and annotation, was done with the CLC Genomics Workbench (QIAGEN, Redwood City, CA, USA). Low quality variants with a score under 200 were filtered out, as well as variants in non-protein-coding regions, synonymous variants, and those present in GnomAD with an allele frequency of over 2%. The remaining variants were assessed for pathogenicity according to ACMG/AMP criteria. The *DICER1* variants were classified as described previously [11].

## Results

### Pathological Findings

**Fig. 2:**
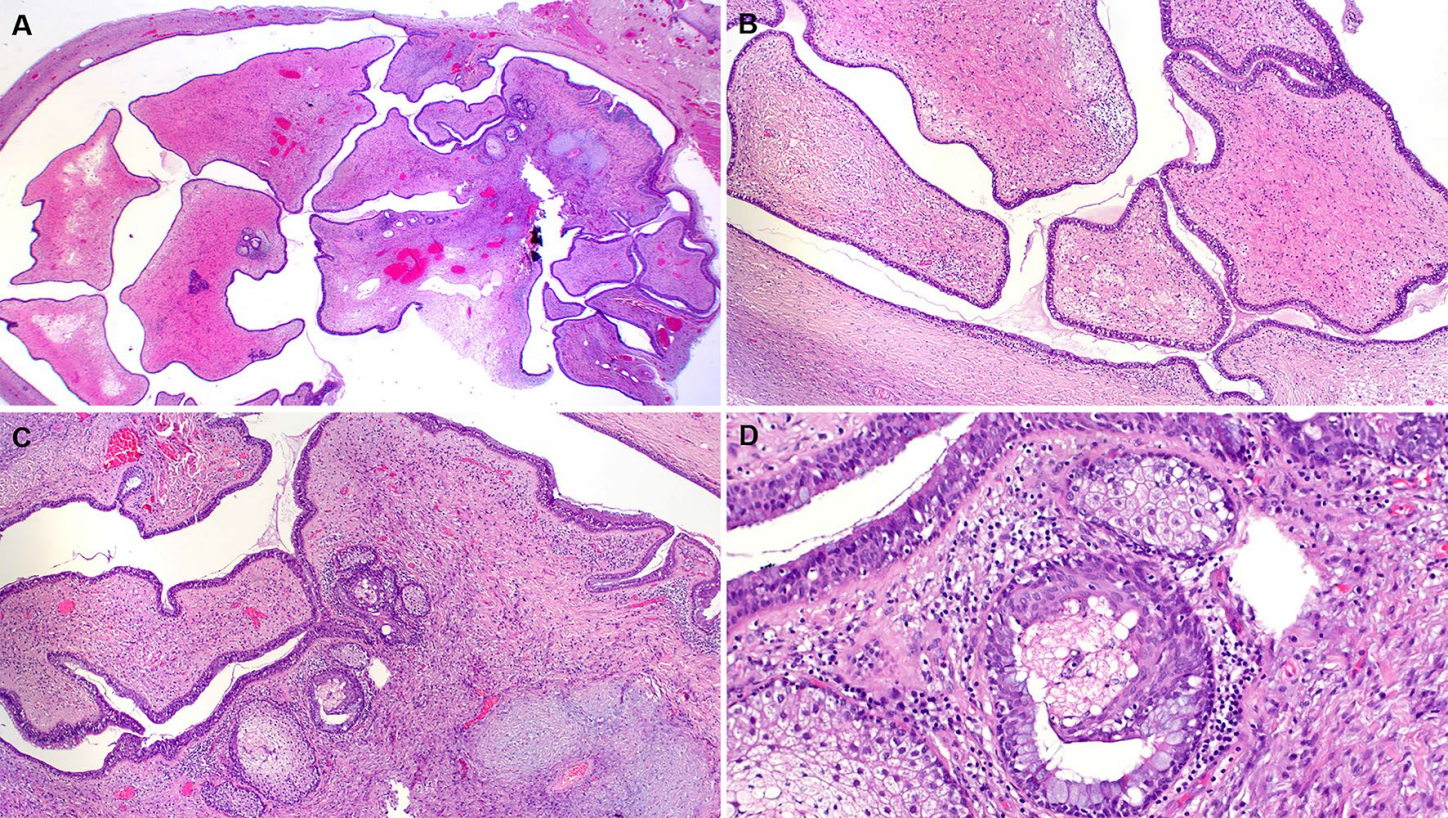
**A** Overview of the histopathological specimen showing phylloides-like fibroepithelial lesion within dilated duct, covered by squamous oral mucosa (upper right field). **B** the lesion was composed of plump edematous to fibrous leaflet-like projections within the cystic cavity, covered by columnar respiratory-type epithelium continuous with the mucosal lining of the cystic duct. **C** Sebaceous elements and focal myxoid change are seen within the stroma. **D** The sebaceous glands merge with the lining epithelium (mid-lower field)

**Fig. 3:**
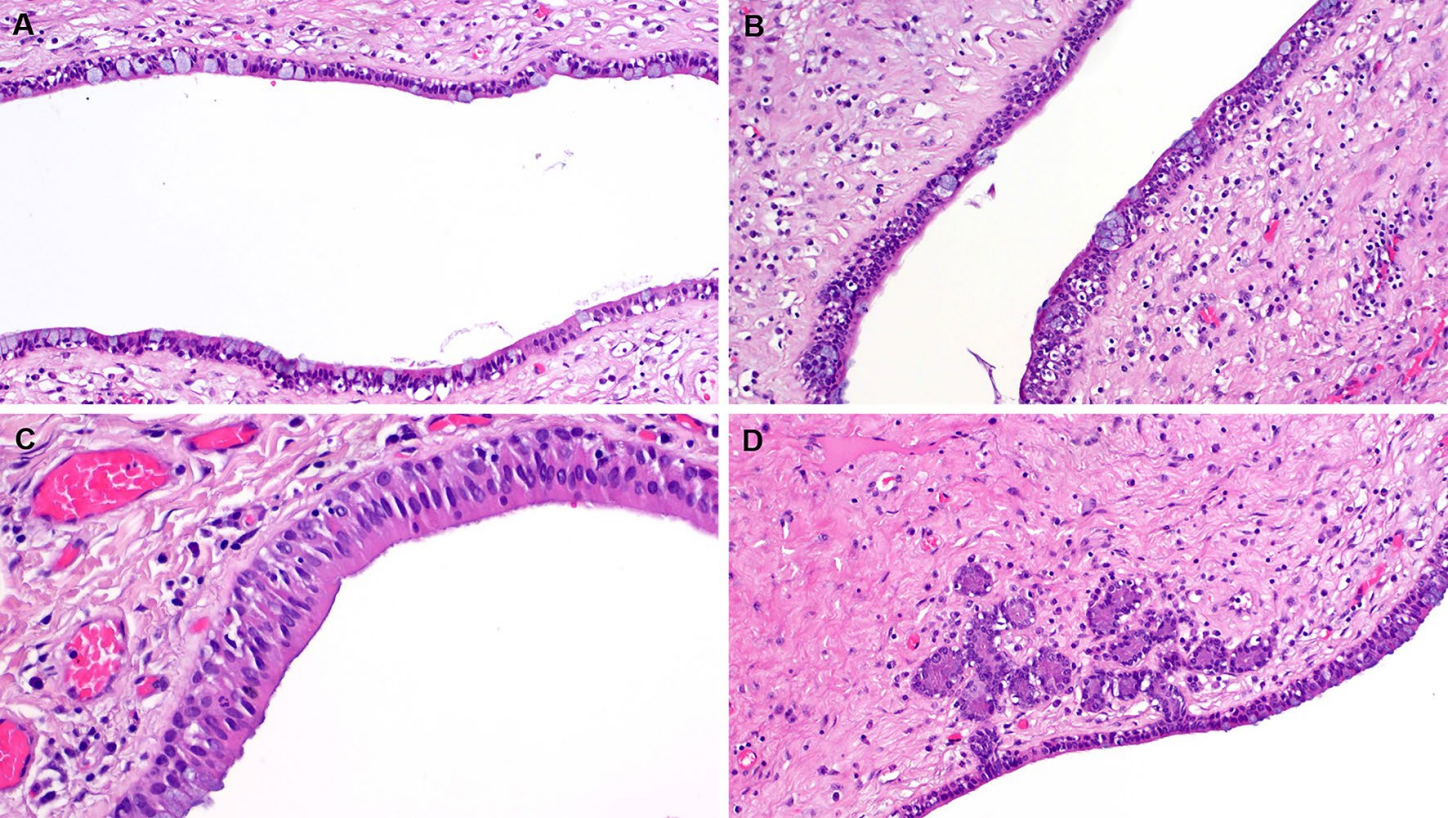
The epithelium lining the cystic duct (upper field) and the epithelial component covering the leaflets (lower field) are more or less similar (**A**), but the latter shows variable hyperplastic changes (**B**, right). **C** The mucus cell-containing epithelium (lower left) merges with eosinophilic columnar ciliated cells lacking mucous elements (**C**). **D** salivary-type serous acini closely associated with the epithelium covering the leaflets

**Fig. 4:**
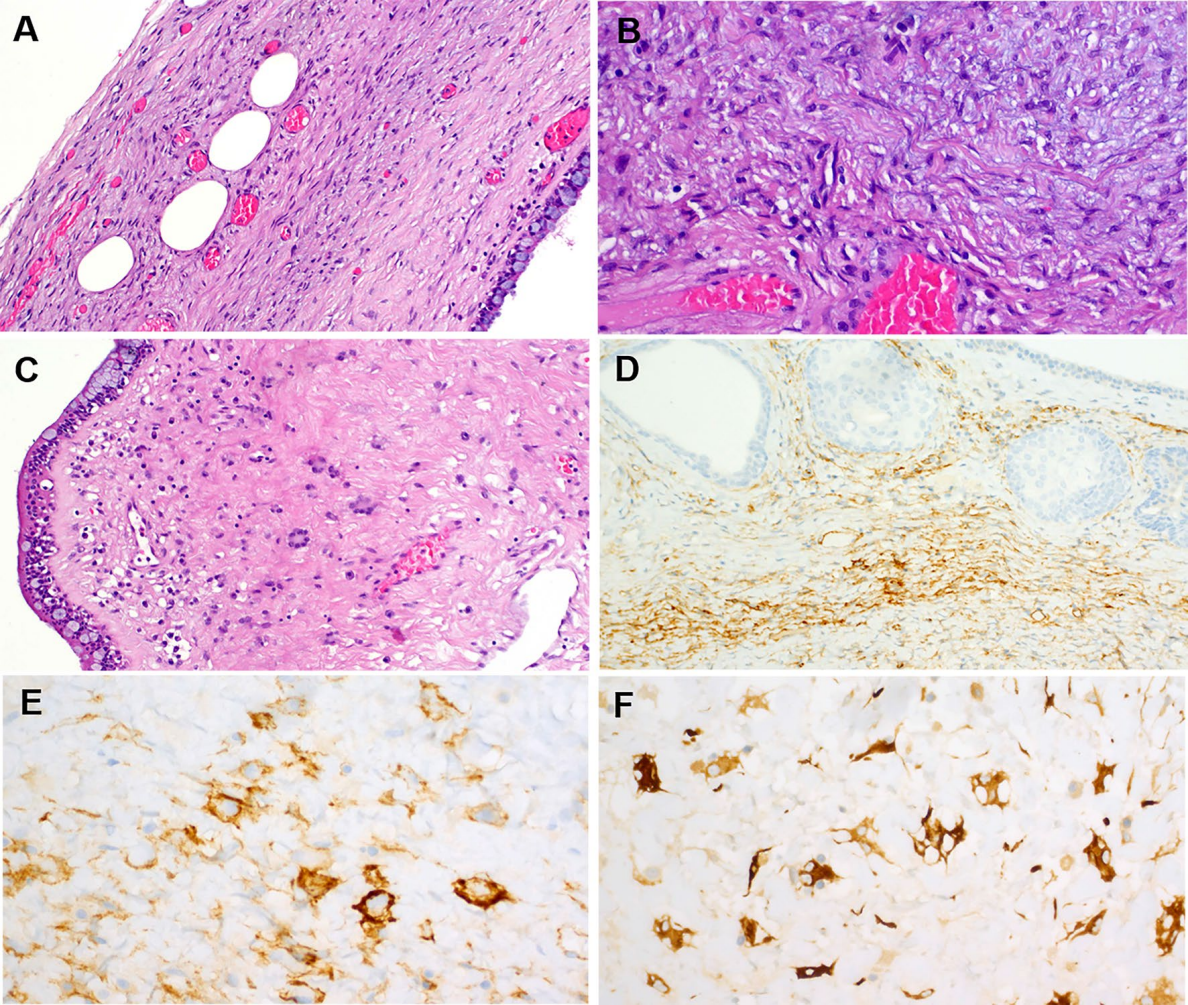
The stromal component was mainly composed of fibroblastic spindle cells entrapping single fat cells at the periphery of the lesion (**A**). **B** higher magnification of the spindle cells, note ectatic vessels. **C** scattered multinucleated stromal giant cells were seen focally. Immunohistochemistry showed experssion of CD34 in the spindle cells (**D**) and the multinucleated giant cells (**E**). Desmin was expressed strongly in the giant cells (highlighting prominent dendritic cytoplasmic processes) and variably in the spindle cells (**F**)

Grossly, the parotid duct showed cystic dilatation measuring 1.5 × 1.0 × 0.9 cm. It was filled with a lobulated soft mass with smooth surface (Fig. 1F). Histologically, the dilated salivary duct was lined by a single layer of columnar epithelium with interspersed goblet cells and non-mucinous columnar cells interrupted by foci of ciliated epithelium without atypia. Within the lumen, there was a large fibroepithelial lesion reminiscent of a phylloides tumor composed of variably edematous or fibrous leaflets covered by similar epithelium as the original duct (Fig. 2A, B). Irregularly distributed sebaceous elements, frequeuntly merging with the respiratory epithelium, were seen beneath the epithelium covering the leaflets (Fig. 2C, D). The epithelium covering the papillary leaflets was similar to and continuous with the luminal epithelium lining the duct but displayed hyperplastic features with increased number of goblet cells and continuous basal cell layer (Fig. 3A-D). Focal aggregates of serous acini were seen within the subepithelial stroma (Fig. 3D), as well as sebaceous elements (Fig. 2C, D). The stroma was moderately cellular, composed of fibroblast-like spindle cells with variable ectatic vessels (Fig. 4A, B), sparse mononuclear inflammatory infiltrates (Figs. 2D & 3B), and focal multinucleated stromal giant cells (Fig. 4C). Foci of micronodular fibromyxoid stromal changes were seen (Fig. 2C). Immunohistochemistry showed a variable expression of CD34 in the spindle cells (Fig. 4D) and multinucleated giant cells (Fig. 4E). Desmin was positive in a few spindled stromal cells with strong expression in the multinucleated giant cells (Fig. 4F). Other markers (STAT6, MyoD1, myogenin, SATB2, S100, MDM2, CDK4, and smooth muscle actin) were negative. Retinoblastoma-1 protein displayed retained expression.

### Molecular Findings

The molecular analysis revealed a *DICER1* mutation (p. [Pro1645fs]; ENST00000343455: c.[4933_4935delCCAinsAG]) with an allele frequency of 7.5%. Sequencing depth at the mutation site was 254×. With a tumor cell content of > 40% within the microdissected and analyzed tissue, the variant appears to be a somatic, heterozygous event. The detected mutation is located within exon 23 of *DICER1* and the affected amino acid is positioned within the functional domain between the RNase IIIa and RNase IIIb domains of *DICER1* [8]. The resulting frameshift affects the functionally crucial RNase IIIb domain suggesting a loss of function effect of the detected variant.

## Discussion

In this case report, we described a “giant” botryoid fibroepithelial polyp of the parotid duct with *DICER1* mutation in an adult female patient. To the best of our knowledge, this is the first documentation of this finding in the salivary glands. Fibroepithelial polyps are polypoid overgrowths of epithelial elements and stromal tissue usually protruding into a hollow organ, a cavity, or overlying a body surface. The epithelial component usually recapitulates the normal epithelium of the organ of origin but may show hyperplastic, metaplastic, or hamartoma-like features. Other mesenchymal elements such as mature skeletal muscle and (rarely) cartilage may be present.

Simple fibroepithelial polyps however are not uncommon. They mainly occur in the skin, where they are also designated as acrochordons or skin tags [12–16]. They also occur in the genitourinary tract and in the oral cavity (predominantly in the buccal area [17, 18]. Rare sites include the orbita [12], larynx [19, 20], tonsil [21, 22], bronchi [23], nasal septum [24], inferior nasal turbinate [25], middle ear [26], and the external auditory canal [27]. Occasinally, these polyps may contain a variable amount of sebaceous glands [28–30]. Fibroepithelial polyps behave mostly in a benign fashion; recurrences after complete excision are rare [31–34].

Large (up to 18.5 cm) fibroepithelial polyps growing into a cauliflower-like (botryoid) pattern have been rarely documented in several anatomical sites [35]. They have been reported in the urinary bladder [10], anus [36] and the nipple [37]. Uncommon sites include the ureter, glans penis, vagina, vulva, perineum and skin. Due to occasional persence of atypical or bizzare-looking stromal cells, multinucleated cells, and myxoid stromal changes, these polyps have historically been termed pseudosarcoma botryoides or pseudosarcomatous polyps [31–35, 38–43]. Grossly and histopathologically, botryoid fibroepithelial polyps may be indistinguishable from botryoid embryonal rhabdomyosarcoma. Hence, their differential diagnosis includes, amongst other lesions, embryonal rhabdomyosarcoma [44], as well as other entities, based on the anatomic location of the lesion [35, 45].

The etiology of fibroepithelial polyps is unknown. A reactive etiology (due to localized trauma or tissue irritation) and a congenital origin have been proposed as potential explanations [46]. Notably, data on genetic findings in botryoid or giant fibroepithelial polyps is scarce. We recently have described one giant botryoid fibroepithelial polyp of the urinary bladder, which showed two non-synonymous *DICER1* pathogenic variants [10]. Reviewing the English literature thoroughly, we were not able to find any more reports on this topic and, to our knowledge, similar botryoid polyps have not been reported in the salivary glands.

Based on its distinctive morphology, we examined the current lesion specifically for *DICER1* mutations. With increasing awareness of their distinctive morphology and the use of modern genetic tools, *DICER1*-associated lesions and neoplasms (both benign and malignant) are becoming increasingly recognized in surgical pathology. These tumors are generally characterized and unified by a biphasic epithelial and mesenchymal growth with the epithelial component essentially reflecting the original epithelium of the organ of origin or its embryonal counterpart, while the stromal component varies greatly based on the tissue of origin, the biology of the entity (whether benign or malignant) and the presence or absence of heterologous mesenchymal tissue derivatives. Accordingly, the patterns seen in many of the *DICER1*-related neoplasms are most likely fibroepithelial or phylloides-like (in benign lesions) or adenosarcoma- or blastoma-like (in malignancies), with some neoplasms showing an admixture of diverse primitive tissue elements resulting in a teratoid appearance. In the head and neck, chondromesenchymal sinonasal hamartoma (mostly hereditary [4]) and thyroblastoma (likely sporadic [47]) represent the most well characterized *DICER1*-related manifestations. Among dental abnormalities and phenotypes associated with the DICER1 syndrome are bulbous crown, periodontitis, and taurodontism [48].

*DICER1* mutations predominantly represent germline variants but may also occur as sporadic (somatic) events. Individuals with the inherited DICER1 syndrome are at increased risk to develop various tumors including pleuropulmonary blastoma [2], Wilms tumor [3], embryonal rhabdomyosarcoma, Sertoli-Leydig cell tumors of the ovary [2], medulloblastoma, cystic nephroma of the kidney, poorly differentiated thyroid carcinoma, intracranial sarcoma, and, more recently identified, pleuropulmonary blastoma-like abdominopelvic sarcoma [49]. Accordingly, the patient’s medical and family history should be checked thoroughly for *DICER1*-suspicious neoplasms. In our case, the *DICER1* pathogenic variant was associated with a low allele frequency, which argued against a germline mutation, consistent with the fact that in the presented case, the patient had no medical or family history of *DICER1*-related lesions.

In summary, we herein describe the first reported case of a botryoid fibroepithelial polyp occurring within the parotid gland duct and carrying a, likely somatic, pathogenic *DICER1* variant. Recognition of this rare presentation is necessary for clinical and genetic work-up of the affected patients. This case nicely highlights the value of standard morphology in predicting the genotype in specific entities which would otherwise have gone unrecognized as being associated with a specific genetic background. Although the current case is likely sporadic, it is important to recognize these rare lesions. Identification of more cases in the future would allow to assess their possible association with the DICER1 syndrome more reliably.
